# Walking All over COVID-19: The Rapid Development of *STRIDE in Your Room*, an Innovative Approach to Enhance a Hospital-Based Walking Program during the Pandemic

**DOI:** 10.3390/geriatrics6040109

**Published:** 2021-11-10

**Authors:** Jaime M. Hughes, John T. Bartle, Ashley L. Choate, Elizabeth P. Mahanna, Cassie L. Meyer, Matthew C. Tucker, Virginia Wang, Kelli D. Allen, Courtney H. Van Houtven, Susan Nicole Hastings

**Affiliations:** 1 Department of Implementation Science, Wake Forest School of Medicine, Winston-Salem, NC 27157, USA; 2 Section on Gerontology and Geriatric Medicine, Division of Internal Medicine, Wake Forest School of Medicine, Winston-Salem, NC 27157, USA; 3 Center of Innovation to Accelerate Discovery and Practice Transformation, Durham VA Health Care System, Durham, NC 27705, USA; ashley.choate@va.gov (A.L.C.); elizabeth.mahanna@va.gov (E.P.M.); cassie.meyer@va.gov (C.L.M.); matthew.tucker1@va.gov (M.C.T.); virginia.wang@va.gov (V.W.); kelli.allen@va.gov (K.D.A.); courtney.vanhoutven@va.gov (C.H.V.H.); susan.hastings@va.gov (S.N.H.); 4 Physical Medicine & Rehabilitation Service, Durham VA Health Care System, Durham, NC 27705, USA; john.bartle@va.gov; 5 Department of Population Health Sciences, Duke University School of Medicine, Durham, NC 27705, USA; 6 Department of Medicine, Duke University School of Medicine, Durham, NC 27705, USA; 7 Duke-Margolis Center for Health Policy, Duke University, Durham, NC 27705, USA; 8 Department of Medicine and Thurston Arthritis Research Center, University of North Carolina at Chapel Hill, Chapel Hill, NC 27705, USA; 9 Center for the Study of Aging and Human Development, Duke University School of Medicine, Durham, NC 27705, USA; 10 Geriatric Research, Education, and Clinical Center, Durham VA Health Care System, Durham, NC 27705, USA

**Keywords:** mobility, activity, hospitalization, rehabilitation, older adults, implementation science

## Abstract

Hospitalization is common among older adults. Prolonged time in bed during hospitalization can lead to deconditioning and functional impairments. Our team is currently working with Department of Veterans Affairs (VA) medical centers across the United States to implement STRIDE (assiSTed eaRly mobIlity for hospitalizeD older vEterans), a hospital-based walking program designed to mitigate the risks of immobility during hospitalization. However, the COVID-19 pandemic made in-person, or face-to-face, walking challenging due to social distancing recommendations and infection control concerns. In response, our team applied principles of implementation science, including stakeholder engagement, prototype development and refinement, and rapid dissemination and feedback, to create STRIDE in Your Room (SiYR). Consisting of self-guided exercises, light exercise equipment (e.g., TheraBands, stress ball, foam blocks, pedometer), the SiYR program provided safe alternative activities when face-to-face walking was not available during the pandemic. We describe the methods used in developing the SiYR program; present feedback from participating sites; and share initial implementation experiences, lessons learned, and future directions.

## 1. Introduction

Older adults are at higher risk for hospitalization compared to their younger peers. Many older adults spend extended periods of time in bed while hospitalized. These periods of extended inactivity can contribute to rapid deconditioning and the onset of new physical disabilities not present upon hospital admission [1,2]. assiSTed eaRly mobIlity for hospitalizeD older vEterans (STRIDE) is an evidence-based walking program aimed at increasing hospitalized patients’ mobility through daily, supervised walks during the course of an inpatient hospital stay. STRIDE began as a clinical demonstration project in 2012 and is currently undergoing widespread implementation in Department of Veterans Affairs Medical Centers (VAMCs) across the United States.

The STRIDE program consists of an initial consult (submitted by a physician or nurse) followed by a gait assessment (typically conducted by a physical therapist) early in a patient’s hospitalization and one to two daily walks supervised by a mobility assistant (e.g., recreational therapy or physical therapy assistant). An initial clinical demonstration evaluation of the program found that older adults participating in STRIDE were more likely to spend fewer days in the hospital and be discharged home rather than a skilled nursing facility [3]. STRIDE is typically implemented on general medicine wards and designed to be flexible in its implementation. For example, sites are encouraged to adapt staffing models, documentation procedures, and marketing tools to maximize “fit” with the local setting.

As of Spring 2020, 18 VA Medical Centers across the United States had implemented STRIDE through a variety of channels including a clinical demonstration project (1 site), regional mandate (4 sites), funding via VA program offices (3 sites), system-wide “Shark Tank” innovation initiatives (2 sites), and participation in a cluster randomized stepped wedge trial sponsored by the VA’s Quality Enhancement Research Initiative (QUERI) (8 sites) [4,5]. All sites were invited to participate in the STRIDE Diffusion Network which was launched in 2018 and facilitated by an interdisciplinary research and evaluation team (“Function QUERI”) comprised of health services researchers, clinicians, and implementation specialists located in Durham, NC. The goals of the Diffusion Network are to capture and share local knowledge and create a collaborative environment for peer-to-peer sharing of experiences and best practices in collaborative quarterly teleconferences. 

The onset of the COVID-19 pandemic created unique challenges and made it difficult for some sites to continue offering STRIDE in-person walks in the face of social distancing and infection control recommendations. This paper describes the evolution and preliminary implementation experiences of STRIDE in Your Room (SIYR), a supplemental effort to support sites in maintaining an inpatient activity program during the COVID-19 pandemic. In this paper, we share the impact of COVID-19 on STRIDE programs and describe the process our team used to develop and evaluate the SiYR program in response to pandemic-related challenges. We offer lessons learned with the goal of using SiYR as one example of how to rapidly adapt and translate program enhancements to help promote activity of hospitalized older adult patients during a national emergency.

## 2. Methods and Materials

### 2.1. Application of Implementation Science Principles

**Figure 1:**
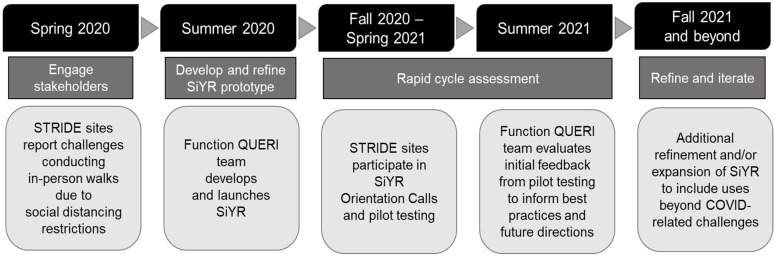
Evolution of STRIDE in Your Room (SiYR) program.

Implementation science focuses on the methods to integrate effective programs into routine clinical practice [6]. Since the onset of the COVID-19 pandemic, there has been growing attention on the value of implementation science in guiding healthcare professionals on how to systematically adapt existing programs in response to pandemic-related challenges [7]. In the spirit of this adaptation, we recognized the need for flexibility in the SiYR program, both to accommodate the changing course of the pandemic and to respect the natural variation that occurs in working with multiple VA facilities across the country. The program described in this paper applied several principles of implementation science, including stakeholder engagement (e.g., local STRIDE program leaders), systematic adaptation, and rapid cycle development and evaluation. Data were drawn from multiple sources, including established data sources integrated into our larger STRIDE program (e.g., quarterly STRIDE Diffusion Network Calls) and additional data sources designed specifically for this project (e.g., quantitative survey and structured email templates). These data sources are described in greater detail below. Figure 1 displays an overview of our development and evaluation process. Below, we describe our multistep process in greater detail, including stakeholder engagement, prototype development and refinement, and rapid cycle assessment.

### 2.2. Engage Stakeholders to Understand the Impact of the COVID-19 on Hospital-Based Mobility

One feature of the national implementation of STRIDE, as described above, is a quarterly Diffusion Network Call. These optional calls are open to all STRIDE sites at any phase of implementation and are designed to serve as a learning collaborative. Calls are structured around one specific topic of interest (e.g., staffing models, data collection, documentation) with ample time for discussion and exchange amongst attendees. In response to feedback received from sites, we dedicated our Spring call held in April 2020 to an open discussion of challenges and experiences related to the COVID-19 pandemic. Below, we report feedback from this call. 

Many VA Medical Centers made changes to facility procedures and clinic workflows upon the initial onset of the COVID-19 pandemic in March 2020. For example, some Medical Centers limited in-person interactions in facility hallways, no longer making supervised STRIDE walks feasible. Additionally, the VA Healthcare System’s fourth mission is to assist the nation in times of emergency, serving both veterans and nonveterans alike [8]. In support of this mission, many VAMCs made preparations in anticipation of accepting higher volumes of suspected and/or positive COVID cases. These preventive steps led to a reduction in many facilities’ overall census (e.g., rescheduling nonemergency procedures to maintain open beds), which, in turn, also reduced the number of STRIDE-eligible patients.

As shared on this call, the impact of COVID-related restrictions varied across sites. Some facilities had limited COVID patients to restricted wards away from other patients, thus having minimal impact on STRIDE walks. Other sites reported facility-level restrictions in hallways. At one site, STRIDE therapists were able to advocate for continuing walks despite these restrictions. However, at least two sites were forced to temporarily suspend their STRIDE programs. As described above, total hospital census was down at all sites. For sites that were not on hold, the reduction in overall hospital census meant potentially fewer eligible STRIDE participants. However, sites also found opportunity in the reduced census. For example, some sites early in the STRIDE implementation process saw the reduced census as an opportunity to dedicate extra time and resources to ramping up their program while other more established programs found an opportunity to expand STRIDE to other wards.

Several sites employed creative strategies to continue keeping patients active despite challenges associated with social distancing and infection control recommendations. At sites where patients were not permitted to leave their rooms, STRIDE staff utilized VA’s Video Connect platform to coach activity sessions over patients’ personal smartphones. However, this required advanced coordination so that a nurse could be present in the room for patient assistance and fall prevention. Other sites relied on existing activity programs that aired via the “GetWellTV Network” available in patient rooms. Despite these innovations, STRIDE sites expressed an interest in identifying more uniform and sustainable solutions to continue walking or exercising safely within the constraints of social distancing and infection control requirements.

### 2.3. Develop and Refine a Prototype

#### 2.3.1. Responding to Stakeholder Feedback

In response to feedback received on our Spring 2020 Diffusion Network Call and in collaboration with the lead STRIDE Physical Therapist (JB), a new solution to enhance the in-patient walking program was developed: STRIDE in Your Room (SiYR). Financial support was made possible through supplemental funding from the VA QUERI program. SiYR was developed in response to four common experiences shared by STRIDE programs during the COVID-19 pandemic:

- Social distancing requirements: At some sites, patients were instructed to remain in their rooms and STRIDE staff were prohibited from supervising face-to-face walks in the facility hallways.
- Rehabilitation support for COVID-positive patients: Patients hospitalized for COVID-19 were challenged by both respiratory symptoms of the virus and physical deconditioning.
- Supplemental tools for STRIDE: Providing patients and STRIDE teams with self-guided exercise could benefit teams during staffing shortages and/or could supplement existing walks for motivated patients who expressed an interest in additional activity.
- Support patients upon hospital discharge: At the height of the pandemic, admission to a rehabilitation and/or skilled nursing facility was complicated by a number of factors. First, some facilities had reduced capacity. Second, facilities often had extended waitlists due to requirements that potential admissions have one or more negative COVID-19 screens. Third, some patients and families preferred to be discharged home out of caution that residential and/or congregate settings may carry increased risk for COVID-19 exposure.

#### 2.3.2. Developing and Refining the SiYR Kit

Overview: SiYR was developed by the interdisciplinary research team with clinical guidance from STRIDE’s principal physical therapist (JB). The format and contents of the kit were also informed by the team’s prior work developing a “STRIDE at Home” program meant to encourage patients to continue walking after leaving the hospital. Finally, feedback from the STRIDE sites (as described above) and safety recommendations from VA Emergency Management and the Centers for Disease Control and Prevention (CDC) also informed the kit.

Kit contents: The SiYR kit was designed to provide hospitalized patients with self-guided activities and light exercise equipment. Together, the components of the kit were designed to promote function and independence via exercises that targeted both large muscle groups and fine motor control necessary to carry out activities of daily living (ADLs). Consideration was given to both natural deconditioning that occurs during a hospital stay and potential respiratory effects of COVID-19. All kit components were developed based on evidence-based activities issued by the National Institute on Aging and COVID-19 safety recommendations issued by the Centers for Disease Control and Prevention (CDC). Together, the components of the kit included self-guided exercises to improve strength and balance, transfer, independent ambulation, activities of daily living, and overall endurance. Kits were designed for individual use and to be sent home upon discharge. All kits were packaged in plastic binders using page protectors that could be wiped down for infection control. Additional details of the kit components are displayed in Table 1.

Pilot testing of initial prototype: After developing the initial prototype, the lead STRIDE physical therapist (JB) completed pilot testing of the SiYR kit over a two-week period at our original STRIDE site in Durham, NC. The goal of this pilot testing was to gain preliminary experience in identifying appropriate patients, introducing the SiYR kit to patients, and documentation of kit distribution. Information gleaned from the pilot testing was then used to develop guidance for other STRIDE sites requesting SiYR kits, including content and instructional materials shared during the SiYR Orientation Calls described below.

### 2.4. Initial Dissemination and Rapid Cycle Assessment with National STRIDE Sites

Launch of SiYR program: The research team introduced the SiYR program to STRIDE sites on a July 2020 Diffusion Network call. The purpose of this call was to describe the different components of the kit and to address any initial questions. A total of 20 participants joined from 7 different STRIDE sites. Call content was made available to sites unable to participate in the call.

Orientation call: All sites requesting SiYR kits were invited to participate in an individual orientation call with the Function QUERI research team and lead Physical Therapist. The orientation call covered the following topics: (1) SiYR purpose; (2) kit contents; (3) identifying appropriate patients; (4) introducing the kit to patients, including how to match activities to patient’s discharge goals; (5) documentation of kit distribution; and (6) sharing feedback with the research team and other STRIDE sites. Although sites were given guidance on how to distribute and document kit use, a primary goal of the orientation call was to remind sites of the freedom involved in kit distribution. Similar to the implementation of the full STRIDE program, SiYR sites were encouraged to adapt the SiYR to best fit characteristics of their individual site, including staffing model and patient needs. The research team emphasized the collaborative, iterative nature of the SiYR program and the potential for each site’s feedback to inform best practices and guidelines for other STRIDE programs implementing SiYR in the future. Finally, the lead physical therapist also shared tips on motivating patients during the pandemic, adopting the phrase “walking all over COVID-19” to highlight the resilience of patients, providers, and staff in rising to the multiple challenges surrounding the pandemic, regardless of COVID status or exposure.

Rapid feedback: As part of the rapid cycle assessment of the SiYR program, three sites volunteered to participate in further pilot testing (*n* = 3), which involved responding to a brief survey at regular intervals (approximately one month and two months after completing the Orientation Call). Survey content is described in Table 2. In addition to these scheduled checkpoints, the Function QUERI team provided all sites with an opportunity to share progress on quarterly Diffusion Network Calls and via structured email communications with the research team. The research team received completed surveys from all three formal “pilot” sites and received feedback from additional sites via email.

## 3. Results

The SiYR program was officially introduced to STRIDE sites in July 2020 on a scheduled Diffusion Network Call (see description above); kit distribution began in September 2020. All STRIDE sites that had implemented a program prior to this date were invited to participate in this supplemental program (*n* = 18). Reach of SiYR was notable with 13 of 18 STRIDE sites (72%) requesting and receiving kits during the program’s initial rollout. Characteristics of participating sites are displayed in Table 3. Participating sites were located in the Central (*n* = 7) and South Atlantic (*n* = 6) regions of the United States. Date of main STRIDE program launch ranged from Fall 2018 to Fall 2020. Just over one-half of participating sites are designated “mid–high complexity” facilities suggesting medium–high volume, medium-risk patients, some complex clinical programs, and medium-sized research and teaching programs [9].

Although participation in SiYR was high, distribution of kits was lower and slower than anticipated across all sites. On average, no site distributed more than five SiYR kits to patients in the first two months of program participation. A summary of feedback is shown in Table 4. Although the SiYR program was not designed as a formal implementation research study, initial feedback provided some indications around implementation outcomes [10]. Qualitative feedback from Diffusion Network Calls suggested that sites found the SiYR kits to be both acceptable and appropriate given limited options to keep patients active in light of social distancing and other COVID-related restrictions. The inherent flexibility of both the original STRIDE program and SiYR, combined with the dynamic course of the pandemic and its variable impacts on different sites within a single healthcare system, made it difficult to evaluate fidelity. Below, we address some lessons learned, including factors that may have impacted sites’ adoption of the SiYR program.

## 4. Discussion

### 4.1. Lessons Learned

#### 4.1.1. Dissemination and Implementation

Although interest in the SiYR was high, kit distribution was slower than anticipated. One of the most common pieces of feedback was difficulty in identifying appropriate patients for the SiYR kit. For patients not already participating in the main STRIDE program (e.g., face-to-face supervised walks), staff expressed concerns with patients having sufficient functional independence to safely participate in an independent activity program like SiYR. Kit distribution may also have been slower as sites were primarily focused on use for patients with a suspected COVID-19 infection or other immunocompromising condition(s). Some sites were impacted by competing clinical priorities, staffing shortages, and/or changes in staffing assignments during the pandemic.

Across all sites, additional guidance on patient identification and SiYR enrollment was desired. More specifically, sites were unclear on when the SiYR kit should be introduced during a patient’s stay and whether patients who decline to participate in the full STRIDE program are still eligible for the SiYR program. Sites also desired more guidance around how to document distribution of the SiYR kit and whether additional uses of the kit should be indicated in the patient’s medical chart.

As described earlier, SiYR was designed to be flexible, similar to the larger STRIDE program. During the Orientation Call, sites were encouraged to adapt both content of the kits and general procedures in distributing and documenting kit use to best fit the needs of their local site. Given the dynamic nature of the pandemic and the variation in COVID-19 cases across geographic regions, our research team was interested in observing how sites might implement a new program during a national emergency. However, our team also recognized the challenges faced by frontline healthcare workers during the pandemic and that, understandably, implementing a new program during this time might feel burdensome and/or less feasible without clear or sufficient guidance. Achieving the right balance between providing sites with clear guidance on how to implement SiYR and allowing flexibility for customization during a pandemic was challenging for the research team.

#### 4.1.2. Optimizing the Kit for Patient Use

STRIDE sites appreciated the tailored exercises provided in the SiYR kit. Some sites had been using established exercise programs (e.g., Otago) which required staff to modify exercises in order to prescribe an activity plan that was appropriate for patients’ functional levels. Although the self-guided exercise booklet was well received by STRIDE staff, sites expressed wanting additional examples that used the exercise equipment provided in the SiYR kit (e.g., TheraBands, foam blocks). Sites also shared that not all exercises were appropriate for patients and that more activity options could be beneficial. Specifically, some patients were too frail for independent exercise included in the SiYR kit while other more active and/or motivated patients could have benefited from more advanced exercises. Finally, some patients had difficulty using the equipment provided in the kit. For those patients not using a mobile phone to track steps, the pedometer’s display was too small to read. For some, utilizing a phone was helpful to track either distance or time walked and use this as a goal to adjust/increase each week. Other patients with dexterity limitations found the foam blocks difficult to use.

#### 4.1.3. Innovation for Rapid Translation

As often shared during the COVID-19 pandemic, innovation arises out of necessity. The inability to safely conduct in-person walks prompted the Function QUERI research team to develop an innovative solution to continue optimizing patient activity in the hospital. Overall, STRIDE sites were eager to adopt the SiYR kit. Feedback from sites suggested that the SiYR kit was comprehensive and an acceptable alternative to face-to-face walks.

However, the pandemic also demonstrated notable variability in impacts on participating sites. Some sites were overwhelmed by high COVID case counts, or regional surges, while other sites experienced lower case counts. Additionally, the ebb and flow of cases over the course of the pandemic also demonstrated a need for flexibility. Although SiYR was designed to be flexible in its initial implementation and to allow for adaptation over time, there were several areas in which sites wanted more direct guidance on how to implement SiYR. It is unknown whether additional guidance, including information on how to engage staff and leadership, may have contributed to wider adoption of SiYR kits.

Beyond brief quantitative surveys and scheduled Diffusion Network Calls, obtaining additional feedback from participating sites was challenging, which may be attributed to a variety of reasons. First, dedicating time to complete quantitative surveys and other administrative tasks may have been difficult given patient care and safety priorities during the pandemic. Second, some sites felt as though they could provide more helpful feedback after distributing more kits. Third, as a rapid cycle improvement project, our research team was focused on addressing a gap in clinical care resulting from the COVID-19 pandemic, not focused on studying a priori research questions with clearly defined metrics of success. This lack of clearly defined goals may have complicated assessment and evaluation.

Despite some of the challenges in distributing kits, some SiYR sites also demonstrated innovation at their local facilities. One team left a SiYR kit on their medical center’s dedicated COVID unit and provided instruction to the unit’s providers on how the kit might be used to promote activity among isolated patients. A second site utilized a “train the trainer” model, educating other facility physical therapists about how to use the SiYR kit and encouraging each therapist to distribute at least one kit. A third site produced a 10-minute video to be played in patients’ rooms that included supine and seated exercises, deep breathing exercises, and information on early mobility during hospitalization. Each of these innovations was presented to the larger STRIDE network as part of quarterly Diffusion Network Calls, further supporting the value of learning collaboratives as an effective implementation strategy, particularly in times of national crises.

### 4.2. Future Directions: Iterative Refinement and Expansion of SiYR

As implementation scientists, our team continues to engage in regular discussion around how the SiYR program can be refined to increase uptake, improve feasibility, and optimize patient use and outcomes. In addition to the lessons learned described above, we highlight potential directions our team may pursue in the future.

#### 4.2.1. Identify Additional Uses of SiYR Kits

Although the future trajectory of the COVID-19 pandemic remains unknown, the introduction of COVID-19 vaccinations has played a major role in slowing new cases. As the pandemic continues to shift, SiYR may be used for other immunocompromised patients who are unable to leave the hospital room and could benefit from a self-guided activity program. SiYR may also be used in times of staffing shortages and/or to supplement existing STRIDE walks. Identifying alternative uses for the SiYR kits may ultimately improve uptake. In addition, providing sites with clear instructions (e.g., written resources, brief training teleconferences) on how to implement SiYR for different uses may also improve uptake and sustainability.

#### 4.2.2. Supplement SiYR Kit with Additional Tools to Support Self-Guided Activity

Whether the SiYR kit is used during a future pandemic or to supplement existing STRIDE walks, our team has identified several tools which may further support self-guided activity. First, patients may benefit from educational and/or motivational text messages both during hospitalization and after discharge. Second, expanding the GetWellTV Network to include brief instructional videos may help patients learn and/or adapt the self-guided activities included in the SiYR kit. Third, postdischarge follow-up calls may be useful as some patients continue to use the SiYR kit after leaving the hospital. Finally, exploring the impact of SiYR on patient-level outcomes will be important in future work.

#### 4.2.3. Partner with Other VA Program and Operations Partners

The VA’s Office of Geriatrics and Extended Care (GEC) has expressed interest in identifying additional uses and/or plans for the dissemination of the SiYR kits. GEC currently leads initiatives to integrate principles of the Age-Friendly Health System [11] into VA settings. SiYR aligns well with one of the Age-Friendly’s 4Ms—mobility.

## 5. Conclusions

The COVID-19 pandemic required our team to think quickly and creatively to identify alternative solutions for helping patients stay active at a time of unprecedented disruption in hospital operations. Inspired by feedback from frontline staff and informed by principles of implementation science, our team launched the STRIDE in Your Room (SiYR) program, an adaption of STRIDE, our existing evidence-based walking program. Although SiYR aimed to address a clinical gap, the challenging and changing circumstances of the pandemic made it difficult for clinical sites to adopt and sustain a new service. We recognize some of the inherent limitations in a rapid cycle assessment project, including implementation within a single healthcare system and prioritization of implementation outcomes over patient-level outcomes. Despite these limitations, lessons learned from this collaborative and iterative process may inform preparedness efforts for future pandemics or other emergency situations. In the meantime, our team will continue efforts to refine the SiYR program and identify how a self-guided exercise kit can serve as a tool to support patient activity both within the hospital setting and upon discharge.

## Figures and Tables

**Table 1:** Components of the SiYR kit.

Component	Description and/or Purpose
Guided Exercises	Self-guided exercises to address weakness, injury, or illness that make it difficult to perform daily tasks. Exercises included those to target large muscle groups, balance, and stability.
Activity Log	Simple log to track daily activity, including minutes of walking, balance activities, strengthening exercise, and/or use of TheraBands or other light exercise equipment.
TheraBands	Three levels of latex-free elastic bands ranging from light to heavy strength (resistance). TheraBands can be used in place of weights for some strength exercises.
Stress Ball	A stress ball can help improve function for picking up items, holding on to objects, and/or performing daily tasks (e.g., eating).
Foam Block	The foam block can be pinched, squeezed, twisted, and moved to improve grip strength and rebuild finger and hand strength.
Pedometer	Pedometers, or step counters, can be helpful in setting goals and tracking daily activity.
Other	Tote bag, pen, STRIDE magnet, hand sanitizer

**Table 2:** Feedback requested from sites during pilot testing.

Domain	Question
Use	What was your main purpose of the SiYR kit?
Recruiting patients	How did you identify appropriate patients? What criteria did you use?
Introducing SiYR to patients	How did you orient patients to the SiYR kit?
Kit components	Which components of the kit were used most frequently?
Successes *	What worked well with the SiYR program?
Challenges *	What challenges did you have?
Suggestions for improvement *	What, if any, suggestions do you have to improve SiYR?
Documentation *	How did you document use of the kit?

* Indicates open-ended response option.

**Table 3:** Characteristics of sites participating in SiYR program.

Site	Region	Facility Complexity Level ^1^	Date STRIDE Program Launched ^2^	Impact(s) of COVID on STRIDE and/or Overall Facility ^3^	Date SiYR Program Launched ^4^	Number of SiYR Kits Requested for Distribution
1	East North Central	1b—High complexity	Spring 2019	STRIDE on hold	September 2020	9
2	South Atlantic	1b—High complexity	Spring 2019	Hallways restricted	September 2020	9
3	East South Central	1b—High complexity	Winter 2018	Reduced census	October 2020	9
4	South Atlantic	1c—High complexity	Spring 2019	-	February 2021	8
5	West North Central	2—Medium complexity	Spring 2019	Reduced census	February 2021	8
6	East North Central	1a—High complexity	Spring 2020	Reduced census	February 2021	8
7	West North Central	1a—High complexity	Spring 2018	-	February 2021 ^†^	8
8	South Atlantic	1c—High complexity	Spring 2019	-	February 2021	8
9	South Atlantic	1a—High complexity	Winter 2019	STRIDE on hold	March 2021	8
10	West North Central	1c—High complexity	Fall 2020	-	May 2021 ^†^	10
11	West North Central	1c—High complexity	Fall 2020	-	May 2021 ^†^	10
12	South Atlantic	1c—High complexity	Spring 2019	-	June 2021	20
13	West North Central	3—Low complexity	Fall 2020	-	June 2021	10

^1^ Designation based on complexity of patient population, complexity of clinical services, and presence of education and research programs [9]. ^2^ Date standard STRIDE program (in-person, face-to-face walking) was launched (Winter, Spring, Summer, Fall). ^3^ Common COVID-related impacts included temporary suspension (hold) of STRIDE program, patients not allowed in facility hallways due to social distancing, and general reduction in overall hospital census. Data are shown for sites participating in Spring or Summer Diffusion Network Calls. ^4^ Defined as date of Orientation Call except where noted (^†^ site declined participation in Orientation Call, start date reflects date kits received).

**Table 4:** Selected feedback received from participating sites.

Domain	Question
Use	Support COVID+ patients
	Provide safe alternative to exercise when social distancing recommendations are in place
	Supplement scheduled STRIDE walks
	Provide additional support upon discharge
Recruiting patients	Able to ambulate independently
	Able to transfer independently
	Cognitively intact
	High motivation
	Patients who may use the kit upon discharge
Introducing the kit to patients	Explain components of kit, demonstrate exercises
Kit components used most frequently	Exercise equipment (TheraBand, foam block)
	Exercise booklet
Successes	STRIDE staff and patients both appreciated the kits
	Kits left with physical therapists (PTs) on COVID unit
	STRIDE PTs instructed other facility PTs on how to use SiYR kits
Challenges	Some patients are too frail to exercise independently
	Higher functioning patients may benefit from more exercises and/or more difficult exercises
	Pedometers were difficult for older patients (e.g., text size too small)
	Low patient compliance after kit was issued
	Exercise log may be overwhelming for some patients
Suggestions for improvement	Guidance on documentation
	Guided exercises for TheraBand and foam block
	More variability in exercises (i.e., option to adapt exercises to patient’s functional abilities)
Documentation	Distribution of kits tracked in external spreadsheet
	Progress note entered into patient medical record

## Data Availability

Data available upon request from the authors.
